# Identifying primary aldosteronism patients who require adrenal venous sampling: a multi-center study﻿

**DOI:** 10.1038/s41598-023-47967-z

**Published:** 2023-12-11

**Authors:** Takumi Kitamoto, Tsuyoshi Idé, Yuta Tezuka, Norio Wada, Yui Shibayama, Yuya Tsurutani, Tomoko Takiguchi, Kosuke Inoue, Sachiko Suematsu, Kei Omata, Yoshikiyo Ono, Ryo Morimoto, Yuto Yamazaki, Jun Saito, Hironobu Sasano, Fumitoshi Satoh, Tetsuo Nishikawa

**Affiliations:** 1 Endocrinology and Diabetes Center, Yokohama Rosai Hospital, Yokohama, 2220036 Japan; 2 Department of Diabetes, Metabolism and Endocrinology, Chiba University Hospital, Chiba, 2608670 Japan; 3 IBM Research, T. J. Watson Research Center, Yorktown Heights, NY 10598 USA; 4 Department of Diabetes, Metabolism, and Endocrinology, Tohoku University Hospital, Sendai, 9808574 Japan; 5 Division of Nephrology, Rheumatology, and Endocrinology, Tohoku University Graduate School of Medicine, Sendai, 9808574 Japan; 6 Department of Diabetes and Endocrinology, Sapporo City General Hospital, Sapporo, 0608604 Japan; 7 Department of Rheumatology, Endocrinology and Nephrology, Faculty of Medicine and Graduate School of Medicine, Hokkaido University, Sapporo, 0608648 Japan; 8 Department of Social Epidemiology, Graduate School of Medicine, Kyoto University, Kyoto, 6048135 Japan; 9 Department of Pathology, Tohoku University Graduate School of Medicine, Sendai, 9808575 Japan

**Keywords:** Endocrinology, Endocrine system and metabolic diseases, Adrenal gland diseases

## Abstract

Adrenal venous sampling (AVS) is crucial for subtyping primary aldosteronism (PA) to explore the possibility of curing hypertension. Because AVS availability is limited, efforts have been made to develop strategies to bypass it. However, it has so far proven unsuccessful in applying clinical practice, partly due to heterogeneity and missing values of the cohorts. For this purpose, we retrospectively assessed 210 PA cases from three institutions where segment-selective AVS, which is more accurate and sensitive for detecting PA cases with surgical indications, was available. A machine learning-based classification model featuring a new cross-center domain adaptation capability was developed. The model identified 102 patients with PA who benefited from surgery in the present cohort. A new data imputation technique was used to address cross-center heterogeneity, making a common prediction model applicable across multiple cohorts. Logistic regression demonstrated higher accuracy than Random Forest and Deep Learning [(0.89, 0.86) vs. (0.84, 0.84), (0.82, 0.84) for surgical or medical indications in terms of f-score]. A derived integrated flowchart revealed that 35.2% of PA cases required AVS with 94.1% accuracy. The present model enabled us to reduce the burden of AVS on patients who would benefit the most.

## Introduction

Primary aldosteronism (PA) is the major cause of secondary hypertension^1–5^. Targeted treatment can mitigate cardiovascular disease in primary aldosteronism^6–8^. More than 50 years have passed since the development of selective central adrenal venous sampling (cAVS) for differentiating unilateral aldosterone-producing adenomas (APA) from bilateral idiopathic hyperaldosteronism (IHA)^9^. The former is surgically curable^6^, while the latter benefits from mineralocorticoid receptor antagonist^7^. The increased use of cAVS to identify surgically curable patients and progress in pathological classification have provided clinicians with a comprehensive understanding of unilateral and bilateral diseases^10–12^. cAVS can only provide unilateral and bilateral laterality in aldosterone secretion^10,12^. However, recent studies on tributary vein sampling using segmental selective AVS (sAVS) have demonstrated that it can distinguish bilateral APAs from IHA, which is classically diagnosed as bilateral PA by cAVS^11,12^. Notably, sAVS can detect APAs in more than 15% of bilateral PA cases compared to cAVS^12^. Therefore, the simplistic view of unilateral or bilateral aldosteronism has become more complicated^13^.

Although significant technical progress has been made, the availability of AVS is still limited in many centers because it is technically demanding. It is unrealistic to assume that AVS will be performed in all PA cases, although many efforts have been made to improve the technical hurdles of AVS^11,14,15^. Therefore, there is a need to identify the subset of patients who require AVS. AVS should be performed to identify APAs for patients with PA who desire to explore the surgical benefits^16^. Since sAVS is more sensitive than cAVS in detecting APAs, it seems more suitable to use the cohort data of PA cases diagnosed by sAVS to develop a prediction model for who should receive AVS.

In developing a prediction model, tumor location and clinical information are crucial. Computed tomography (CT) is a reasonable choice for determining location information because of its ease of accessibility, as shown in prior studies^17–19^. However, CT has two limitations: (1) tumors < 6 mm in diameter are undetected^20^, and (2) inability to distinguish from other types of adrenocortical tumors. Therefore, clinical markers representing the distinctive pathophysiological characteristics should be identified to overcome the limitations of CT in predicting APAs. Our understanding of the pathophysiological characteristics of APAs has progressed significantly, owing to the discovery of somatic mutations in the genes encoding *KCNJ5*^21^, *ATP2B3*, *ATP1A1*^22^, *CACNA1D*^23^, *CACNA1H*^24^, *CLCN2*^25^, and *CTNNB1*^26^. These aldosterone synthesis driver mutations account for over 90% of APAs^27^. Among them, *KCNJ5* mutation has a crucial pathophysiological role in APAs; the most frequent somatic mutations are evident in clinical characteristics, such as young age, female sex, progressive autonomous aldosterone production with suppressed renin, severe serum hypo-potassium, and large tumors^28^. In addition to these clinical features, the responsiveness of aldosterone secretion to adrenocorticotropic hormone (ACTH), indicated by the ACTH stimulation test (AST) and dexamethasone suppression test (DST), was distinctive in APAs harboring *KCNJ5* mutation^29–32^. We focused on these clinical markers, which may reflect significant pathophysiological characteristics of APAs.

It is often challenging to obtain certain clinical markers in some centers because of resource constraints. The key question in practical subtyping is how a prediction model trained on a reference dataset from one center applies to *other* centers that may have missing data in a particular way. To date, all published studies on sAVS have been conducted in a *single*-center cohort^11,12,14^, and none have addressed this critical issue in practice.

This study aimed to establish a practical approach to distinguishing between patients with PA affected by APA and those affected by IHA. For this purpose, we study the CT-guided subtyping approach in a *multicenter* setting where sAVS is available, and the diagnoses of the surgically treated cases were confirmed through pathology and post-surgical follow-up^6^. We develop a machine learning-based CT-guided subtyping prediction model with well-established sAVS cohort data^12^ as the training data, using clinical markers that potentially reflect the pathophysiology as the predictor variables. To address the issue of cross-center data heterogeneity, we develop an approach called the adaptation–classification framework. Specifically, before applying a classification model trained on the reference sAVS cohort dataset, we adapt each center to the reference center using a probabilistic data imputation model. Based on the trained subtyping model, we established a clinical flowchart for identifying cases that require AVS.

## Results

### Diagnostic outcome of the multi-center cohort of PA cases diagnosed by sAVS

This study was conducted over three institutions, Sapporo City General Hospital (Sapporo), Tohoku University (Sendai), and Yokohama Rosai Hospital (Yokohama), where sAVS was available for PA diagnosis. We used a previously published and well-established cohort^12^ as reference data (or training data; *N* = 278) on which the adaptation and classification models were trained. A multicenter cohort (Yokohama, Sapporo, and Sendai; *N* = 210 in total) was used as validation or test data for the adaptation-classification framework. Further details on the data are provided in the “Methods” section.

The diagnostic outcomes and clinical characteristics of the 210 patients with PA in the multicenter cohort are shown in Table 1. A total of 89 and 121 patients were diagnosed with uni- and bilateral PA, respectively. Of 121 patients with bilateral PA, 13 were diagnosed with bilateral APAs and underwent surgery to alleviate their symptoms. The remaining 108 patients with IHA were treated with medication. Surgically treated patients demonstrated significantly higher plasma aldosterone levels and lower serum potassium levels than those in the IHA group. Among patients with unilateral PA, 92.1% and 33.7% achieved postsurgical biochemical and clinical cures, respectively. The PA cases in Sendai presented the most severe clinical phenotype, whereas those in Sapporo showed the mildest phenotype with more IHA cases (Tables S1–3). The low consistency between the cAVS and sAVS (Table S4) was similar to the prior study^12^. This is due to differences in plasma cortisol concentrations between both adrenal sides in sequential sampling^33^, resulting in diagnostic outcomes of cAVS without ACTH stimulation, which tends to show unilateral cases. Cannulation stress also affects plasma cortisol secretion. Therefore, this difference can be eliminated by ACTH stimulation^12^ or by simultaneous sampling from each side by inserting two catheters^33^. As it is not feasible to perform simultaneous sampling from every tributary vein on both sides and we wanted to minimize the invasion caused by catheter insertion, we used sequential sampling in the present study. sAVS could identify more of the 26 cases with surgical benefits than those identified through the conventional approach using the lateralization index. However, using only CT in this cohort would have misled the surgical indication in 50 of the 210 cases (23.8%): 38 would have received surgery on the wrong side, and 12 who would have benefitted from the surgery would not have been identified (Table 1).

**Table 1:** Comparison of clinical characteristics among patients diagnosed with unilateral or bilateral primary aldosteronism treated with surgery or medication.

Variables	Unilateral PA	Bilateral PA		*P* value
	Surgery	Surgery	Medication	
	(*n* = 89)	(*n* = 13)	(*n* = 108)	
Sapporo, Sendai, Yokohama	15/33/41	1/1/11	41/39/28	
Age (yr)	51.9 ± 11.2	51.3 ± 11.9	49.9 ± 11.2	0.4657
Sex (male/female)	51/38	6/7	40/68	0.0177
SBP (mmHg)	143.9 ± 16.6	141 ± 18.5	142.2 ± 19.5	0.7477
Plasma aldosterone (ng/dl)	37.7 (23.5–59.8) †	41.1 (22–51)‡	17.8 (13.9–24.2)†‡	< 0.0001
Plasma renin activity (ng/ml/hr)	0.2 (0.1–0.3) *†	0.4 (0.2–0.6)*‡	0.3 (0.2–0.5)^†‡^	0.0024
Lowest serum potassium ion concentration (mmol/L)	3.2 ± 0.6^†^	3.3 ± 0.4^‡^	3.7 ± 0.4^†‡^	< 0.0001
Resected adrenal nodule size (mm)	13.7 ± 7.7	11.6 ± 7.8	N.A	0.3584
Laterality of surgical side (right/left)	44/45	10/3	N.A	0.0789
Laterality of image positive side (right/left/bilateral/undetectable)	30/39/14/6	5/2/5/1	7/16/9/76	< 0.0001
Diagnostic outcome of cAVS (right/left/bilateral/failed)	38/39/9/3	8/3/1/1	41/13/51/3	< 0.0001
L.I. in cAVS	17 (3.8–35.9) †	9.6 (2.5–17.5) ‡	2.2 (1.4–4.5)^†‡^	< 0.0001
Diagnostic outcome of ACTH‐cAVS (right/left/bilateral/failed)	34/35/18/2	4/3/6/0	0/0/108/0	< 0.0001
L.I. in ACTH‐cAVS	9.8 (5.1–23.7) *†	4.2 (1.7–7.5)*‡	1.4 (1.2–1.8)^†‡^	< 0.0001
Biochemical outcome (complete/partial/absent) [complete (%)]	82/5/2 [92.1%]	7/6/0 [53.9%]	N.A	< 0.0001
Clinical outcome (complete/partial/absent) [complete (%)]	30/53/6 [33.7%]	2/11/0 [15.4%]	N.A	0.1993

The clinical characteristics and diagnostic outcome of the present multi-center cohort. Technical cannulation failures occurred in five cases in the right adrenal vein before ACTH stimulation, and two cases after ACTH stimulation. Unilateral sAVS results and the laterality of CT-detectable tumors were used for their diagnosis. *, ^†^, ^‡^Significantly different pairs. Data are expressed as mean ± standard deviation or median (interquartile range). SBP, systolic blood pressure; DBP, diastolic pressure; cAVS, central adrenal venous sampling; L.I., lateralization index; ACTH, Adrenocorticotropic hormone; N.A., not applicable.

### Clinical characteristics of the cases requiring AVS

**Figure 1:**
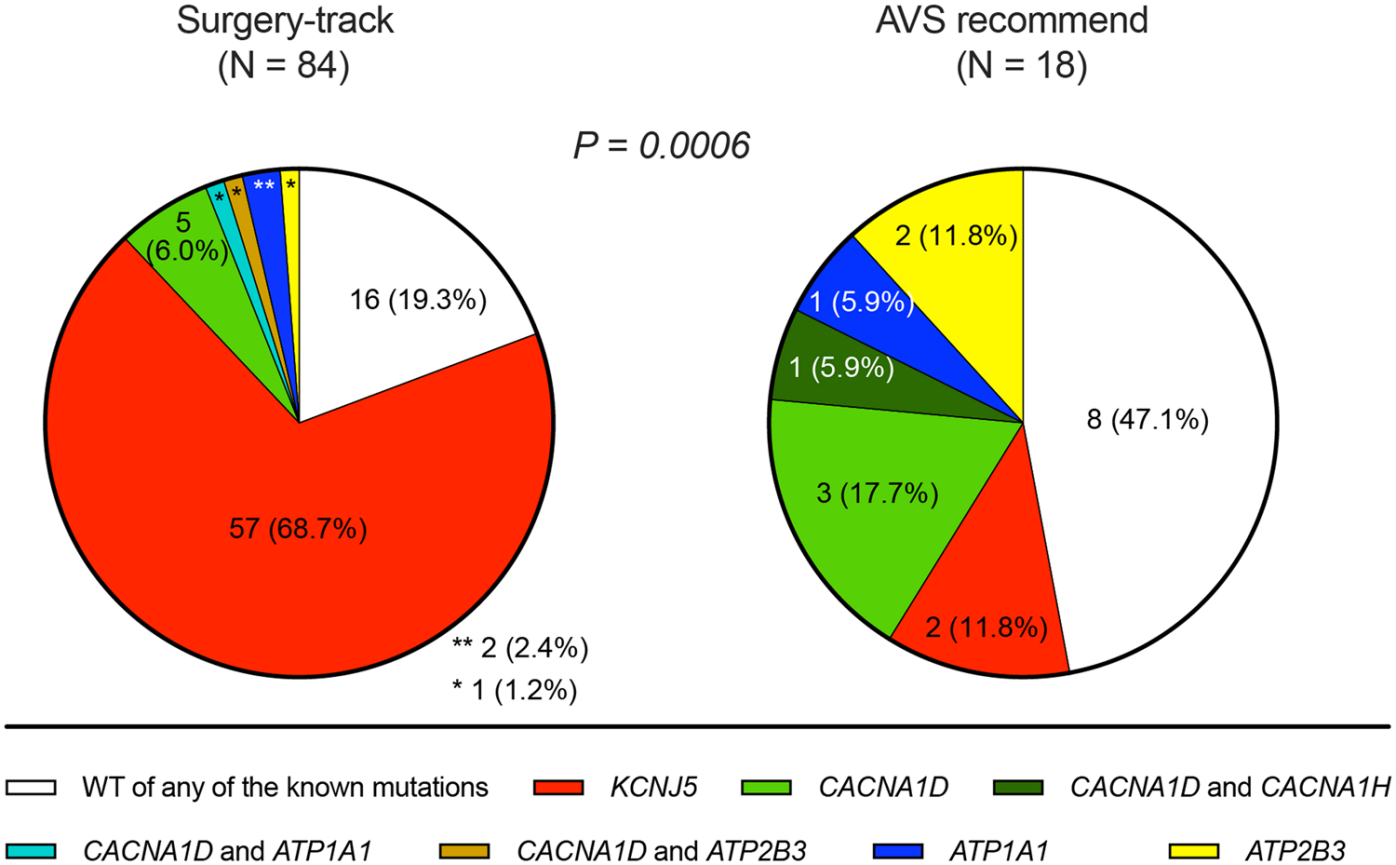
The distribution of aldosterone driver gene mutation identified in APAs. The number and proportion of each mutation is shown. Two samples from Sendai were not available in this analysis. Yates’ chi-squared test was applied to detect significance.

To develop a CT-guided prediction model for PA cases requiring AVS, we retrospectively defined three categories as follows: “surgery-track” (APA identifiable as a tumor visible on CT), “AVS-recommended” (APA undetected on CT), and “medication-track” (to be medically treated for IHA diagnosis) (see the detail in the “Methods” section). We performed gene sequencing of resected APAs for aldosterone driver mutations in *KCNJ5*, *ATP2B3*, *ATP1A1*, *CACNA1D*, and *CACNA1H* to understand their pathophysiological characteristics. The results demonstrated a skewed distribution of *KCNJ5* (68.7% vs. 11.8%) and *CACNA1D* mutations (8.4% vs. 23.5%) (p = 0.0006) (Fig. 1). These data indicate distinct molecular pathogeneses in the present categories. Patients in the surgery track were younger, predominantly women, had a higher ARR on CCT, had larger tumors, and had better clinical outcomes than those in the AVS-recommended group (Tables 2 and S5).

**Table 2:** Comparative characteristics of clinical markers between surgery-track vs. AVS recommended group.

	*N*	Surgery-track	AVS recommend	*P* value
		84	18	
Sex (male/female)	102	42/42	15/3	**0.0168**
Age (yr)	102	50.5 ± 11.1	57.7 ± 10.1	**0.0131**
Duration of HT (yr)	100	10 (4–17.3)	6.5 (3–16.5)	0.5177
BMI (kg/m^2^)	102	24.3 ± 3.6	23.7 ± 3.7	0.5461
Antihypertension medication (defined daily dose)	101	2 (1–3.3)	2 (1.2–3.1)	0.883
SBP (mmHg)	102	142.6 ± 17.1	147.9 ± 14.8	0.2206
DBP (mmHg)	102	88.7 ± 12.8	89 ± 13.4	0.9324
Plasma aldosterone (ng/dl)	102	40.4 (23.7–58.8)	27 (18.6–55.6)	0.1879
Plasma renin activity (ng/ml/hr)	102	0.2 (0.1–0.3)	0.3 (0.2–0.4)	0.0621
Serum cortisol (μg/dl)	95	8.2 (6.5–10.7)	9.3 (7.5–12.4)	0.2205
Serum cortisol after DST (μg/dl)	91	1.1 (0.7–1.6)	1 (0.5–1.4)	0.3946
Serum potassium (mmol/L)	102	3.2 ± 0.5	3.3 ± 0.5	0.3273
Cases treated with potassium replacement	102	56 (66.7%)	11 (61.1%)	0.6548
Serum creatinine (mg/dl)	102	0.8 ± 0.3	0.8 ± 0.2	0.6136
eGFR (mL/min/1.73 m^2^)	102	79.4 ± 24.4	75.2 ± 14	0.4817
ARR after CCT	100	105.8 (62.6–214)	55.9 (38.0–151.6)	**0.0258**
Plasma aldosterone after AST (ng/dl)	95	67.2 (37.9–91.1)	46.1 (29.6–64.6)	0.1106
Plasma cortisol after AST (μg/dl)	95	22.2 (19.9–25.4)	26 (21.6–28.5)	**0.0167**
Laterality of image positive side (right/left/bilateral/undetectable)	102	34/37/13/0	1/4/6/7	** < 0.0001**
Adrenal nodule (mm)	102	15.7 ± 6.2	3.2 ± 5	** < 0.0001**
sAVS diagnosis (unilateral/bilateral)	102	74/10	15/3	0.6962
Laterality of surgical side (right/left)	102	45/39	9/9	0.7829

The clinical parameters used for a model to identify the cases in need of AVS. The number of the available samples is shown in the column of *N*. The data presentation is shown in the same way as Table 1. The Defined daily dose is the assumed average maintenance dose per day for a drug used for its main indication in adults. HTN, hypertension; DST, dexamethasone suppression test; eGFR, estimated glomerular filtration rate; CCT, captopril challenge test; AST, ACTH infusion test.

Significant values are in bold.

### Developing adaptation model

We used 36 clinical markers as the predictor variables for subtype prediction, derived from AST, DST, and CCT, which may reflect responsiveness to ACTH or Renin-Angiotensin, tumor information obtained from CT-imaging (see the detail in the “Methods” section “Training classifiers”) besides demographic and common biochemical data. The proposed framework consists of two modules: adaptation and classification. The adaptation module captures the most informative subspace of the predictor variables from the reference data and is used to fill in the missing data of the multicenter cohort. Mapping the samples onto the same subspace enables domain adaptation between the reference and the multicenter cohorts. For the classification module, we compared three well-known classifiers: logistic regression (LR), random forest (RF), and multi-layer perceptron (called deep learning (DL) hereafter). These classifiers were trained on the reference data and applied to the multicenter cohort after missing fields were imputed with the adaptation module. The details of model training are provided later in this study.

Figure S1 illustrates how the missing fields were imputed compared with the naïve mean imputation approach. Because of our probabilistic formulation, different patients received different imputed values depending on their observed attributes and values.

**Figure 2:**
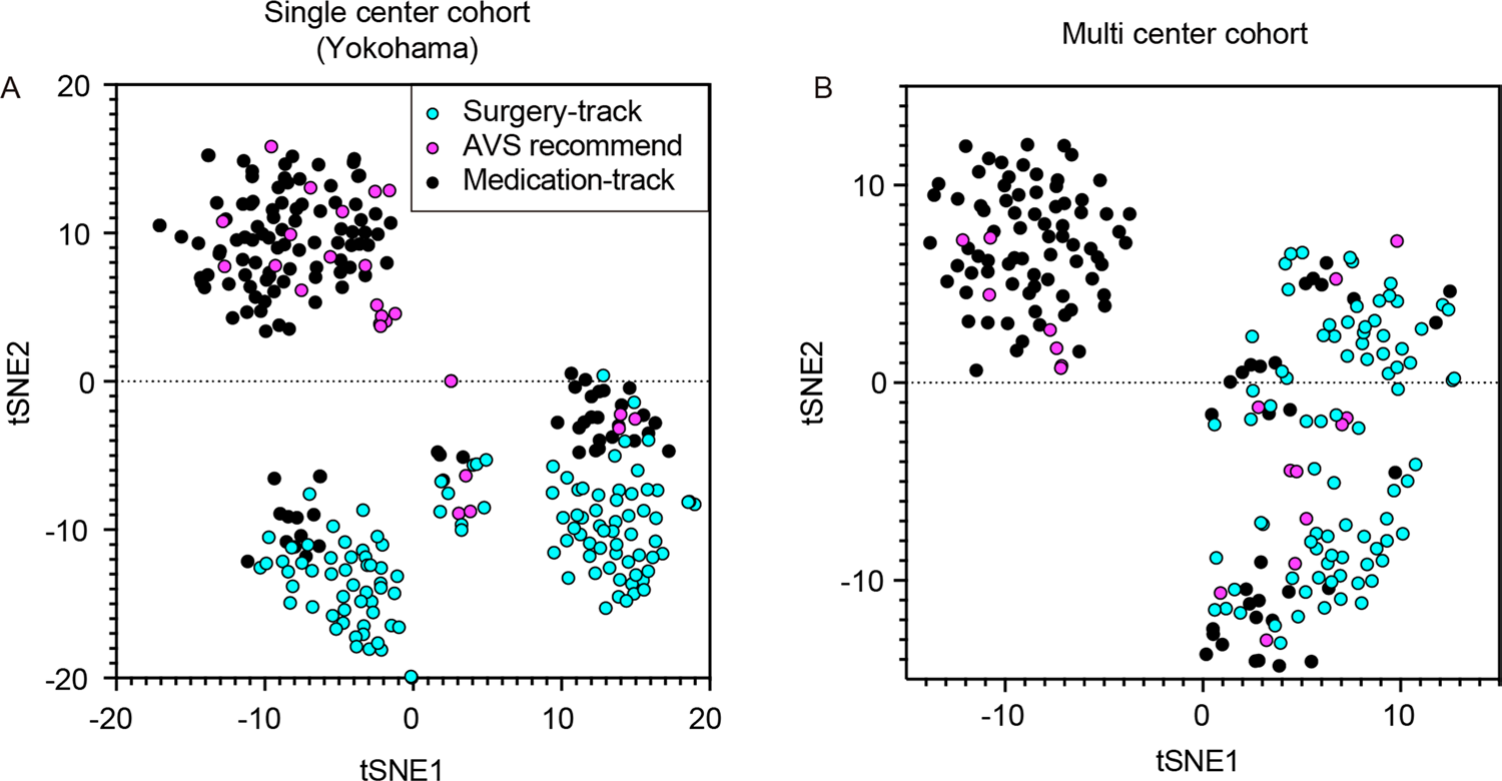
*t*-SNE plot of the cases in single and multi‐center PA cohort. *t*-SNE plot used clinical characteristics of the cases in the single-center^12^ (**A**), and the present multi-center cohort (**B**). This analysis have done after having the missing fields of each data-set imputed. The class-wise distributions had many commonalities between single vs. multi-centers. Cyan circle: Surgery-track; Magenta circle: AVS recommend; black circle: Medication-track.

In Fig. 2, we visualize two cohorts with *t*-distributed stochastic neighbor embedding (*t*-SNE) after imputing the missing fields. The figure shows that class-wise distributions have many commonalities between the reference and multicenter cohorts, suggesting that the CT-guided case identification approach is applicable across different centers. The figure also shows that the second category (“AVS-recommended”) is scattered across multiple clusters, suggesting binary classification would be a more reasonable strategy than three-class classification.

### Developing classification model

Guided by the distribution presented in Fig. 2, we trained two binary classifiers. The first was to distinguish the surgery track from the others, and the second was to distinguish the medication track from the others. In either case, we computed the sensitivity (true positive ratio (TPR)) and specificity (true negative ratio (TNR)), and used the f-score^34^ as the harmonic mean between the TPR and TNR as the main performance metric (see the supplemental material for details).

**Figure 3:**
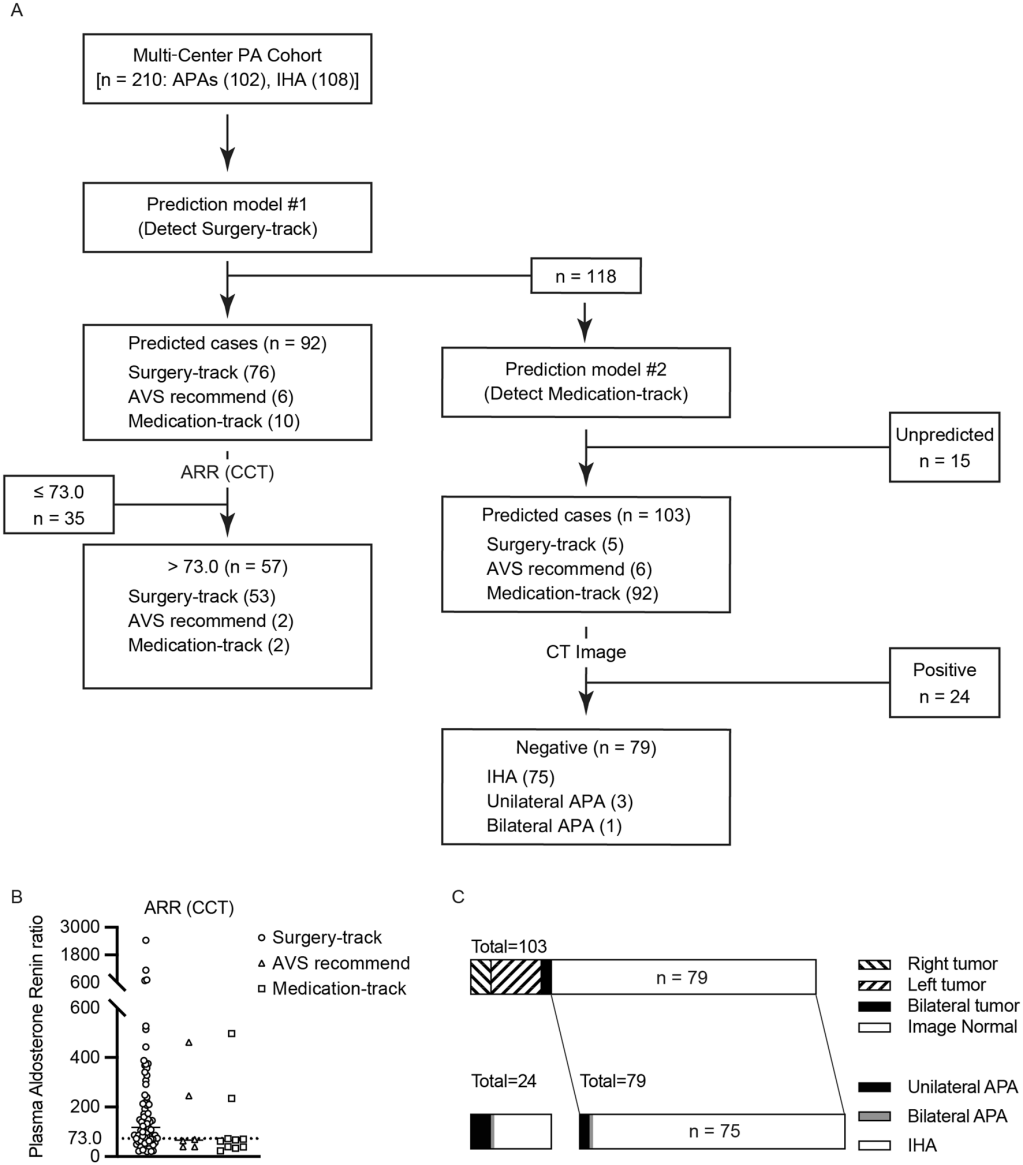
Machine learning model assistant diagnostic strategy of PA patients. (**A**) Diagnostic flow chart using the present models; the actual number of each category is indicated. We first used the prediction model for surgery track, resulting in 92 candidates for surgery, and not for 118 cases. For the candidates for surgery, we applied a cut-off value of more than 73.0 of ARR in CCT, we could narrow down 57 candidates for surgery. The remained 35 cases needs to receive AVS for their surgical indication. We applied the prediction model for medication track for 118 cases, resulting in 103 candidates for medication. The remained 15 cases need to receive AVS to determine surgical indication. Among 103 candidates for medication, we could focus on CT negative cases to select 79 cases to be provided medication. The remained 24 cases should receive AVS to rule out surgical indication. Therefore, 35, 15 and 24 cases (74 cases as total) should receive AVS to determine their treatment strategy. (**B**) The comparative distribution of ARR after captopril challenge test (CCT) in Surgery-track, AVS recommend, and Medication-track. The cut‐off value is indicated in dashed line. (**C**) The comparative results of CT findings in the predicted cases by Medication-track model.

The results are summarized in Table 3. The f-scores ranged from 0.82 to 0.89 and 0.84 to 0.86 in the surgery-track and medication-track models, respectively. The predictive performance of each model was higher for LR than for RF and DL (f-score: Surgery-track, 0.89 vs. 0.84, and 0.82; Medication-track, 0.86 and 0.84 vs. 0.84). The LR coefficients, which approximately correspond to the importance of the variables, are shown in Figs. S2 and S3. Plasma aldosterone and renin levels, tumor size, and estimated glomerular filtration rate (eGFR) were positive predictive factors in the surgery-track group, whereas negative CT findings were negative predictive factors (Fig. S2). In contrast, negative CT findings and serum potassium levels were positive predictive factors for the medication track, whereas plasma aldosterone levels were a negative factor (Fig. S3). We generated a diagnostic flowchart for clinical use using the two LR models (Fig. 3a). The surgery-track model predicted 92 cases and provided surgical indications with CT results for 57 cases, of which 53 (93%) were in the surgery-track group, for cases with an ARR of more than 73.0 after CCT (Fig. 3b). Of the other 118 cases, 103 were predicted to be IHA using the medication-track model. When the model was applied to image-negative cases, 79 cases were identified, of which 75 (95%) were IHA (Fig. 3c). Overall, our sequential flowchart identified that 35.2% of patients with PA required AVS, with an accuracy of 94.1%. Table S8 shows detailed clinical information on four cases in which the diagnostic flow led to a recommendation for surgery in cases with AVS recommendation or on the medication track. Two surgical cases involved bilateral tumors, with a small tumor considered as the dominant autonomous aldosterone source, and a postoperative biochemical cure was obtained. One drug-treated patient showed a severe PA phenotype with progressive renal dysfunction and was diagnosed with IHA with cortisol-producing adenoma. The other medically treated patient was diagnosed with bilateral PA using sAVS, for which surgery was not performed.

**Table 3:** The comparison among diagnostic machine learning models.

	Sensitivity	Specificity	f-score	Hyperparameters
Surgery-track				
Logistic regression model (L2 regularization)	0.90	0.87	0.89	(balanced, C = 4, penalty = l2)
Logistic regression model (L1 regularization)	0.89	0.82	0.85	(balanced, C = 1, penalty = l1, solver = liblinear)
Random Forest	0.87	0.81	0.84	nTrees = 2000
Deep learning	0.75	0.88	0.82	(Nh0, Nh1, Nepoch, batch) = (6,5,100,8)
Medication-track				
Logistic regression model (L2 regularization)	0.87	0.85	0.86	(balanced, C = 0.005, penalty = l2)
Logistic regression model (L1 regularization)	0.89	0.81	0.85	(balanced, C = 0.11, penalty = l1, solver = liblinear)
Random Forest	0.87	0.81	0.84	nTrees = 2000
Deep learning	0.82	0.86	0.84	(Nh0, Nh1, Nepoch, batch) = (6,5,100,16)

Each model included all 36 covariates, which are listed in Figs. S1 and S2. Surgery- and Medication-track models are shown. The results provide accuracy of prediction for each category with f-score values and set parameters in each machine learning method.

## Discussion

Our machine learning-assisted diagnostic flow identified that 35% of PA cases required AVS. The flow can reduce the burden and economic cost of the PA diagnosis process for the patients. In addition, the effort would achieve the generalization of definitive PA diagnosis by navigating patients with PA to specialized referral centers according to their benefit from AVS. The present referral centers had different extents of specialty and displayed etiological outcomes consistent with those of a previous study^4^. Similar to a recent multicenter international cohort^35^, one-third of the PA cases showed negative CT images. This consistency suggests that the selection bias of the present multicenter cohort was minimal, if any, and that the model can be extended to realistic patient data comprising an imperfect dataset. The advantages of the present model are as follows: (1) Using the cohort identifying a more significant number of surgically treatable PA cases than those identified by the conventional AVS approach, (2) the model can be applied for cases without a complete dataset by the transfer learning technique to impute missing values, and (3) the model can predict not only APA cases that can benefit from surgery but also identify PA cases that should be treated with medication.

Leveraging our unique imputation technique and machine learning-based classifiers, 36 common clinical markers, including CT image information, were used to develop a model for multicenter cohorts. Previous studies that developed AVS bypass models categorized both unilateral and bilateral cases. Only a few robust clinical markers that showed statistically significant differences in multivariate analysis were integrated into the model^18,19,36–42^, and only three studies were conducted with a multicenter cohort^38,39,41^. In these studies, one study showed that four out of 58 cases predicted as unilateral (6.9%) received adrenalectomy on the wrong side as a CT-visible tumor located on the wrong side^18^, and the others did not mention this point clearly. The present multi-center cohort showed that CT-detectable tumors mislead laterality in 50 out of 210 PA cases, similar to a recent international multi-center study^43^ [23.8 vs. 28(%)] (Table 1). Thus, predicting unilateral versus bilateral cases is insufficient in clinical settings. Information on the side that should undergo adrenalectomy is also required. In addition, owing to the high proportion of discordance between visible tumors and laterality, the diagnostic outcome needs to be validated using postsurgical outcomes. Unfortunately, only two prior studies were available on postsurgical biochemical PA resolution in their cohorts^19,39^. Our cohort addressed these points, and we labeled the cases according to the treatment strategy, which was surgery track, medication track, or AVS-recommended instead of labeling uni- vs. bilateral PA. Using our model for surgical indications, we determined the laterality of CT-detectable tumors for surgery-track cases; otherwise, the cases were classified as the AVS-recommended group.

In the proposed framework, the adaptation module plays an important role in ensuring the practical utility of the proposed approach. As confirmed by our observations in the present study, missing data patterns are highly center-specific. In small medical institutions, obtaining hundreds of fully observed samples and training prediction models are challenging. We addressed this challenge using a transfer learning technique and reused the latent principal subspace learned in the data-rich reference center to regularize the multicenter cohort. Adaptation was performed such that the estimated data distribution fitted the observed data fields as much as possible. Although transfer learning has recently gained popularity, specifically in medical imaging^44,45^, most studies have focused on reusing the neural network parameters of a pretrained model. Little work has been conducted to address these particular issues in multicenter settings.

In this study, we used predictor variables commonly measured across various institutions. This implies that these variables have relatively strong support for being informative when predicting the outcomes. These carefully selected variables were expected to produce a relatively simple classification boundary, as shown in Fig. 2. In this case, the high model capacities of DL and RF can be redundant, resulting in no positive improvement in the prediction performance.

We found that *KCNJ5* somatic mutations were almost exclusively found in APA tissues in the surgical track group, indicating their unique pathological mechanisms. *KCNJ5* mutation has a remarkably high frequency in Asian PA cases^29,46–52^. The present study detected the *KCNJ5* mutation in 59% of APAs, while a previous Japanese single-center cohort reported it in 69.4%–78.7% of cases^50,53,54^. This difference can be attributed to the heterogeneous etiology of PA. Owing to the superior interpretability of LR, we reaffirmed the crucial role of three significant factors, PAC, serum potassium level, and tumor size, in the prediction of surgery and medication tracks (Figs. S2 and S3). Moreover, responsiveness to renin–angiotensin–aldosterone and ACTH-cyclic 3’,5'-adenosine monophosphate signaling pathways, represented by CCT and AST, contributed to the model following the three major factors. When new molecular markers for *KCNJ5*-mutated APAs^55,56^ become available, the present model can be updated.

Our algorithm enabled the identification of patients who could benefit from AVS. In other words, it predicts the suitability of surgical or pharmacological therapies for diagnosis at a level comparable to that of the sAVS. It also distinguishes cases in which prediction is challenging; that is, patients who require AVS to explore the benefits of surgical intervention. Our findings indicate that AVS is necessary in 35% of patients (PA). Using the results of this study, 65% of patients could bypass the AVS procedure using our classifier, allowing for early treatment. Furthermore, this model relies on information obtained from common clinical indicators and CT images available across institutions, even when data are missing. This universality is a significant strength for its practical implementation in clinical settings.

The present study has the following limitations: (1) it was conducted retrospectively in endocrinology units; (2) AVS diagnostic criteria were not the same among the institutions; (3) all the PA cases evaluated in the study were Japanese, who are known to show a specific etiology of somatic mutation prevalence^52^; and (4) sAVS is available only in a limited number of centers^11,12,14,15^. We should note that cAVS missed the surgical benefit in 25% of cases identified as requiring AVS in the present model. To address this issue, we have been taking action to distribute our sAVS technique internationally^11,12,14,15^. Future studies should be conducted prospectively using the same diagnostic procedures applied in all cases. Moreover, the results should be evaluated in Western countries and other Asian populations in addition to the Japanese population.

In conclusion, we developed a reasonably accurate prediction model to determine AVS requirements for therapeutic strategies in patients with PA. Furthermore, our model has the advantage of being transferable to real-world patient data. The developed clinical flowchart can be used for the therapeutic decision-making process of physicians and patients. This will allow AVS to be provided to patients with PAs.

### Perspectives

The present study was designed to pursue the maximum possibility of bypassing sAVS in a CT assistant prediction model using widely available clinical factors across multiple centers. Our model identified 35.2% of the patients needing sAVS with 94% accuracy. However, 4 out of 210 cases (1.9%) could not be categorized and were falsely indicated to undergo surgery, and the other four cases missed surgical treatment. The four cases falsely indicated for surgery were unique (Table S8). As shown by the two cases that required AVS for diagnosis, there is still room for improvement in predicting the responsible lesion in cases with bilateral tumors. The effectiveness of surgical intervention in the two cases classified as "Medication-track" is not clear for patients with IHA who also have strong aldosteronism, advanced complications, or left–right differences. Even if AVS is available, the clinical judgment is complex.

We assume that this gap was not due to the study design, including cohort characteristics and pattern recognition strategies, as we leveraged the best knowledge to date. As introducing new imaging technologies generally costs more than biomarkers, a combination of upcoming biomarkers that reflect pathophysiological signatures would enhance our model. The enrichment of *KCNJ5* mutations in the sAVS bypass group may play an important role. Biomarkers that are highly specific to APAs harboring *KCNJ5* mutations or IHAs complemented the current model. Future studies in this field will focus on integrated locational and pathophysiological prediction models to identify patients who require sAVS.

## Methods

### Third party material

All of the material is owned by the authors.

### Study design and participants

This study was conducted in Sapporo City General Hospital (Sapporo), Tohoku University (Sendai), and Yokohama Rosai Hospital (Yokohama), where sAVS was available for PA diagnosis. The study received overall institutional approval [the research ethics committee of Yokohama Rosai Hospital (30-100)] and site institution approvals [the ethics committee of Sapporo City General Hospital (R01-059-573) and Tohoku University School of Medicine (2019-1-274)]. The participants provided written informed consent. Research had been performed in accordance with the Declaration of Helsinki. They retrospectively included consecutive PA patients diagnosed between 2015 and 2017 in this study. All participants had a definitive diagnosis of PA during hypertension screening based on the Japan Endocrine Society (JES) guidelines^57^. Pathological diagnosis and post-surgical biochemical outcomes were evaluated to validate the diagnosis.

We used previously published data^12^ as the reference cohort (or training data; *N* = 278) to train the adaptation and classification models. The present multi-center cohort data was used as the test or validation data (*N* = 210). It should be noted that training and test/validation data are separated by design in this setting, eliminating the need for manual data splitting when evaluating the performance against the multi-center cohort.

### Diagnostic procedure for primary aldosteronism and concomitant subclinical Cushing’s syndrome

According to the JES guidelines^57^, the antihypertensive drugs prescribed for the patients were changed to budralazine, α-blockers, or calcium channel blockers several weeks before blood sampling. Mineralocorticoid antagonist were replaced 4 weeks before evaluation, and other medications were 2 weeks before the test. A 30-min rest in the supine position preceded the morning blood sample collection. Plasma aldosterone concentration [PAC (ng/dl)], serum cortisol concentrations [F (µg/dl)], and plasma renin activity [PRA(ng/ml/hr)] were measured using specific radioimmunoassays (RIA). The PAC and active renin concentrations, as evaluated by chemiluminescent enzyme immunoassay, were converted to PAC and PRA using RIA for comparison as previously reported^58^. We used a PAC/PRA ratio (aldosterone-to-renin ratio [ARR]) > 20 as the PA screening criterion and performed one or more confirmatory tests (captopril challenge test [CCT], furosemide-upright test, or saline-loading test) to confirm the presence of PA^57^. We evaluated a 1-mg overnight DST to detect subclinical Cushing’s syndrome.

### Subtype definition

As mentioned before, we retrospectively defined three categories to identify PA cases requiring AVS as follows: “surgery-track” (APA identifiable as a tumor visible on CT), “AVS-recommended” (APA undetected on CT), and “medication-track” (to be medically treated for IHA diagnosis), which makes the subtyping problem a three-class classification problem. We retrospectively assigned the surgically treated cases with bilateral tumors into the surgery-track group if their APAs are located on the larger tumor side; otherwise, they were assigned into the AVS-recommended group. Because tumor size is the only clue to determine the surgical laterality for PA cases with bilateral tumors, and resected tumor dominantly exists in the larger tumor side according to AVS diagnosis. Therefore, those in surgery-track, they can perform adrenalectomy for the site of the larger tumor side, while those in the AVS-recommended group require AVS to determine their surgical indication.

### Training classifiers

We selected common clinical variables used in the PA diagnostic process for the classifiers, for which missing data were observed in less than 20% of each institution. Notably, we obtained tumor information from the CT images for the classifier as follows: (1) tumor size: right and left tumor size, (2) tumor laterality: right-sided, left-sided, bilateral, or image-negative, and (3) larger tumor size and laterality. For example, in the case of bilateral tumors, 5 mm on the right side and 8 mm on the left side, the input parameters were as follows: (1) right 5 mm tumor and left 8 mm tumor, (2) bilateral, and (3) 8 mm tumor and left side. The size of the image-negative tumor was set to zero. As a result, we have 36 clinical markers, which were used as the predictor variables in patient subtyping (the variables are shown in Figs. S2 and S3).

As previously discussed, maintaining cross-center consistency is one of the biggest challenges in developing a prediction model in the multicenter setting. There were two main sources of data heterogeneity: (1) calibration issues for clinical markers and (2) missing data. Although a common set of clinical markers has been carefully selected and calibrated across multiple centers to address the former, the latter is unavoidable for various center-specific reasons, including the availability of medical resources.

To address this issue, we developed an approach called the adaptation–classification framework. Specifically, we used the previously reported^12^ well-managed single-center data as the reference dataset (or the “source domain” in the terminology of transfer learning) to train a domain adaptation model as well as the classifier. In our framework, domain adaptation is performed by imputing missing data using the probability distribution of the reference data. We developed a new variant of Bayesian principal component analysis (BPCA), which can be viewed as a lightweight version of the state-of-the-art variational autoencoder (VAE)-based data imputation algorithm^59^ and is particularly suitable when only a limited number of samples are available. One major advantage of our BPCA algorithm is that it is virtually parameter-free, and hence has a minimal risk of overfitting, which is in sharp contrast to deep-learning-based approaches. In particular, it automatically determines the dimensionality of the principal subspace. A detailed description of the algorithm is provided in the Supplementary Material.

For comparison, we trained three well-known classification algorithms: logistic regression (LR), random forest (RF), and multilayer perceptron (called deep learning (DL)). These algorithms were trained in a binary classification setting, where the samples of either the surgery track or medication track were treated as positive samples. The objective functions to be minimized were the negative log-likelihood for LR, the Gini index for RF, and binary cross-entropy for DL. The training was performed using the standard open-source software packages: LR and RF used scikit-learn 1.0, and DL used Keras 2.4.0. Hyperparameters such as the number of trees in the RF, were chosen via a grid search, so the f-score was maximized on the validation data. Details of the model training are provided in the Supplementary Material.

### Statistical analyses

JMP^®^ 16 (Statistical Analysis System Institute Inc., Cary, NC) was used for statistical analyses. Variables with normal or non-normal distributions were expressed as mean ± standard deviation (SD) or median (interquartile range [IQR]). The Student’s *t*-test or the Mann–Whitney U test was used for comparisons between groups. One-way analysis of variance or the Kruskal–Wallis test was used for multiple comparison tests. The significant differences among the groups were determined using Tukey’s post hoc analysis or the Steel–Dwass analysis. The relative proportions of categorical variables were assessed using Yates’ chi-squared test or Fisher’s exact test. Statistical significance was set at P < 0.05.

### Supplementary Information

Supplementary Information.

## Data Availability

The data that support the findings of this study are available from the corresponding author upon reasonable request. Extended methods are provided in the supplemental material.

## Abbreviations

AVS: Adrenal venous sampling
PA: Primary aldosteronism
sAVS: Segmental selective AVS
IHA: Idiopathic hyperaldosteronism
cAVS: Central AVS
APA: Aldosterone-producing adenoma
CT: Computed tomography
RIA: Radioimmunoassay
PAC: Plasma aldosterone concentration
PRA: Plasma renin activity
ARR: Aldosterone-to-renin ratio
CCT: Captopril challenge test
ACTH: Adrenocorticotropic hormone
AST: ACTH stimulation test
DST: Dexamethasone suppression test
LR: L2-regularized logistic regression
RF: Random Forest
DL: Deep learning
BPCA: Bayesian principal component analysis
MAP: Maximum a posteriori
*t*-SNE: *T*-distributed stochastic neighbor embedding
SD: Standard deviation
IQR: Interquartile range

## Code availability

The source code will be made available on Github (https://github.com/Idesan/bpca) upon the acceptance of the paper.
