# Supplementary material for: Identifying primary aldosteronism patients who require adrenal venous sampling: a multi-center study﻿
Source: Sci Rep. 2023 Dec 11;13:21722. doi: 10.1038/s41598-023-47967-z (PMC10713522; doi:10.1038/s41598-023-47967-z)
Supplement: Supplementary file 1 — Supplementary Information. [file 41598_2023_47967_MOESM1_ESM.pdf]

Identifying Primary Aldosteronism Patients who Require Adrenal Venous Sampling: A Multi-center Study

Takumi Kitamoto, MD, PhD[1, 2 †, *], Tsuyoshi Idé, PhD[3, †], Yuta Tezuka, MD, PhD[4, 5], Norio Wada, MD, PhD[6], Yui Shibayama, MD, PhD[6, 7], Yuya Tsurutani, MD, PhD[1], Tomoko Takiguchi, MD, PhD[1], Kosuke Inoue, MD, PhD[8], Sachiko Suematsu[1], Kei Omata, MD, PhD[4, 5], Yoshikiyo Ono, MD, PhD[4, 5], Ryo Morimoto, MD, PhD[5], Yuto Yamazaki, MD, PhD[9], Jun Saito, MD, PhD[1], Hironobu Sasano, MD, PhD[9], Fumitoshi Satoh, MD, PhD[5, 9], and Tetsuo Nishikawa, MD, PhD[1]

[1] Endocrinology and Diabetes Center, Yokohama Rosai Hospital, Yokohama, Japan, 2220036,

[2] Department of Diabetes, Metabolism and Endocrinology, Chiba University Hospital, Chiba, Japan, 2608670, [3] IBM Research, T. J. Watson Research Center, Yorktown Heights, NY, USA, 10598, [4] Department of Diabetes, Metabolism, and Endocrinology, Tohoku University Hospital, Sendai, Japan, 9808574, [5] Division of Nephrology, Rheumatology, and Endocrinology, Tohoku University Graduate School of Medicine, Sendai, Japan, 9808574, [6] Department of Diabetes and Endocrinology, Sapporo City General Hospital, Sapporo, Japan, 0608604, [7] Department of Rheumatology, Endocrinology and Nephrology, Faculty of Medicine and Graduate School of Medicine, Hokkaido University, Sapporo, Japan, 0608648, [8] Department of Social Epidemiology, Graduate School of Medicine, Kyoto University, Kyoto, Japan, 6048135, [9] Department of Pathology, Tohoku University Graduate School of Medicine, Sendai, Japan, 9808575

Supplemental material

This appendix has been provided by the authors to give additional information about the present study.

Contents

Extended Methods

Table S1. Comparison of clinical characteristics in Sapporo related to Table 1

Table S2. Comparison of clinical characteristics in Sendai related to Table 1

Table S3. Comparison of clinical characteristics in Yokohama related to Table 1

Table S4. The concordance of diagnostic outcomes among sAVS, cAVS before and after ACTH stimulation, and CT imaging

Table S5. Post-operative clinical parameters are shown in Table 2

Table S6. Primer pairs used for mutation detection by Sanger sequencing

Table S7. Summary of the classifiers trained

Table S8. Clinical characteristic of the cases mis-classified into Surgery-track

Figure S1. Data imputation of the single dataset and transfer learning between two datasets

Figure S2. Breakdown list of the coefficients from the logistic regression model to detect the cases on the surgery-track

Figure S3. Breakdown list of the coefficients from the logistic regression model to detect the cases on the medication-track

Extended Methods

Blood Pressure Measurements

Measurement and clinical evaluation of BP were according to the Japanese Society of Hypertension guidelines [1]. Office BP values were obtained in a sitting posture using a standard upper arm blood pressure monitor after a 5-minute rest. The average of three consecutive values was recorded. Blood pressure measurement was done by a fully automated oscillometric sphygmomanometer in a quiet place.

Adrenal venous sampling

sAVS was conducted to determine the intra-adrenal localization of hyperaldosteronism. The details of the procedure have been described in a previous study [2-4]. We performed thin-section computed tomography (CT) scans of the adrenal glands using a contrast medium. The success of catheter insertion into the adrenal veins was confirmed based on the selectivity index (SI), which was defined as the ratio of the adrenal cortisol concentration divided by that in the peripheral vein ([cortisol]adrenal vein/[cortisol]peripheral vein). The cut-off value of SI was ≥2.0 before and ≥5.0 after ACTH stimulation[5,6]. The LI was calculated with the samples from the central veins, that is ([aldosterone]/[cortisol])-dominant adrenal vein/([aldosterone]/[cortisol]nondominant adrenal vein). For the comparative analysis among CT images, cAVS, and sAVS, we adopted the cut-off value of LI ≥2.0 before ACTH stimulation and ≥4.0 after stimulation[7]. The absolute plasma aldosterone value was also used for hyper aldosterone secretion with a threshold of ≥1,400 ng/dL[3]. Regarding sAVS, Sapporo and Yokohama mainly used absolute plasma aldosterone values, while Sendai mainly used LI ≥2.6 to define the lesion causing hyperaldosteronism[2]. In Sapporo, sAVS was primarily performed for cases with CT detectable tumors.

DNA preparation, genotyping, and mutation detection

Tissue samples from patients with APA were frozen in liquid nitrogen immediately after excision and stored at -80°C before use. Genomic DNA was prepared from tumor tissues by the standard procedure described previously[8,9]. Sanger sequencing for the mutation hot spot of the *KCNJ5*, *ATPase*, *CACNA1D*, *CACNA1H,* and *CTNNB1* was performed using the primers shown in Table S6. In Sendai, APA samples without *KCNJ5* mutation by Sanger sequencing were analyzed using targeted next-generation sequencing to determine other somatic mutations as previously described[9].

Evaluation of postsurgical outcomes

Biochemical and clinical assessments were performed 6–18 months after surgery. We categorized these outcomes as per the description in the Primary Aldosteronism Surgery Outcome study[10]. Briefly, the normal blood pressure was defined based on the Eighth Joint National Committee (JNC8) guidelines[11]. Patients were categorized as having achieved complete, partial, or absent clinical success based on the postsurgical changes in blood pressure and antihypertensive medications. Moreover, patients were categorized as achieving complete, partial, or absent biochemical success based on the correction of hypokalemia and normalization of the plasma aldosterone/renin ratio (ARR). We evaluated the confirmatory tests of patients with high ARR.

Classification module

[Training-test separation] As discussed in the main text, there are two different datasets in our problem. One is the well-established reference dataset from a previously published work. The other is the multi-center cohorts, which contain data samples from three centers (Sapporo, Sendai, and Yokohama) and may have a center-specific pattern of missing data. Upon training a

classifier, the former was used as the training dataset and the latter was used as the test/validation dataset. It should be noted that training-test separation is made by design, and there is no need for manual data splitting in this setting.

[Classification algorithms] For comparison purposes, we trained three classifiers—logistic regression (LR), random forest (RF), and a multi-layer perceptron (called deep learning (DL) hereafter). In modern machine learning classification algorithms, LR is known for its superior interpretability, where fitted values of regression coefficients can be directly used as the degree of importance. In contrast, RF and DL are considered as black boxes, while they can handle nonlinear classification boundaries and potentially have higher predictive performance. This is a well-known trade-off between interpretability and accuracy that must be considered when choosing an algorithm [12]. Another important dimension of the model choice is robustness. DL is efficient when millions of samples are available; however, it may overfit a small dataset owing to its irregular classification boundary.

[Performance metric] We cast the original three-class classification problem into two binary classification problems, guided by the observations from Fig.2, where either surgical-track or medication-track was defined as the positive class. As the main performance metric, we used the harmonic mean between the true positive ratio (TPR; sensitivity) and the true negative ratio (TNR; specificity):

$$f = \frac{2 \times \mathrm{TPR} \times \mathrm{TNR}}{\mathrm{TPR} + \mathrm{TNR}}$$

We call this the f-score in this study, following Ilicic et al[13]. This score was used also as the metric to choose the best set of hyperparameters.

[Model parameter optimization approaches] Table S7 summarizes the objective functions and hyperparameters tuned. For LR, we used either L1- or L2-regularized model, and the model parameters were optimized on the training dataset with the coordinate descent algorithm as implemented as ‘liblinear,’ which is a natural extension of the standard IRLS (iteratively reweighted least squares) algorithm [14]. The hyperparameter (‘C’) is the inverse of the regularization strength and was grid-searched to maximize the f-score on the validation dataset (i.e., the multicenter cohorts). For the other hyperparameters, we used the default values of scikit-learn 1.0: The solver was ‘liblinear’ with the maximum number of iterations of 100; The class weights were adjusted with the number of negative and positive samples (‘balanced’). For RF, the number of trees (‘ntrees’) was grid-searched against the f-score evaluated on the validation dataset. The other hyperparameters were set to the default values of scikit-learn 1.0: The minimum samples per leaf was 1; The minimum samples per split was 2; The maximum features searched in partitioning was 6; Bootstrapping was performed when creating the trees. For DL, we employed a multi-layer perceptron with two hidden layers. The activation function of those hidden layers is ReLU (rectified linear unit), while the sigmoid function was used in the output layer. The number of neurons of the hidden layers (‘Nh0’, ‘Nh1’) and the batch size (‘batch’) were grid-searched against the f-score evaluated on the validation dataset. As the number of test samples is as small as 210, we fixed the learning rate to a relatively small value, 0.001, which was confirmed to produce a smooth learning curve and was combined with a relatively large maximum epoch (‘Nepoch’) of 100. The other hyperparameters were set to the default values of Keras 2.4.0: The optimizer was Adam; Early stopping and regularization were not used, considering the low dimensionality of the predictor variables.

Adaptation module

The main functionality of the adaptation module is to impute missing data of a newly acquired *test* dataset. It is done by using the probabilistic distribution of predictor variables leaned from *the training data*. The applicability to a new dataset is its critical feature.

Bayesian principal component analysis (BPCA) is a well-known data imputation approach, first proposed by Oba et al.[15] While it has been used in a variety of application fields for years as one of the state-of-the-art imputation methods[16], most of the existing works do not provide a method for out-of-sample (i.e., test sample) extension. Therefore, we proposed a new BPCA variant that enables out-of-sample imputation, emphasizing the automatic relevant determination (ARD) [17] for determining the latent dimension *d*.

The Bayesian generative model expressed below was used in the study.

$$p_0(x \mid z) = \mathrm{Normal}(x \mid Wz + \mu, \epsilon \mathrm{I}_M),$$

$$p_1(z) = \mathrm{Normal}(z \mid 0, \mathrm{I}_d),$$

$$p_2(\mathrm{W} \mid \alpha_1, \dots, \alpha_d) = \prod_{l=1}^{d} \mathrm{Normal}(w_l \mid 0, \alpha_l \mathrm{I}_M),$$

where x is the vector of M predictor variables, "Normal" is the normal (or Gaussian) distribution, and p_0 is the Gaussian observation model with a mean $Wz + \mu$ and a covariance matrix $\epsilon \mathrm{I}_M$. Here, W is a $M \times d$ matrix whose column space spans the principal subspace, and z is a d-dimensional latent vector to be estimated that can be viewed as a "cleaned" version of x, which generates the observable x through the linear mapping by W. In addition, p_1 is the prior distribution for z, while p_2 is the prior distribution imposed on each column vector of $\mathrm{W} = [w_1, \dots, w_d]$, where the l-th column vector of W is denoted by w_l , which is an M-dimensional vector. Here, $\mathrm{I}_M, \mathrm{I}_d$ are the M- and d-dimensional identity matrices, respectively. ϵ is assumed to be a given constant while the variances $\{\alpha_1, \dots, \alpha_d\}$ are learned from the training data. As discussed below, d is typically set to M and lets ARD choose the dimensionality.

Suppose we have a training data set containing N samples $\{x^{(1)}, \dots, x^{(N)}\}$, which are assumed to be standardized to ensure that the mean and the variance of each dimension are zero and one, respectively. Under the above model, each sample $x^{(n)}$ is associated with a latent vector $z^{(n)}$, whose prior distribution is given by $p_1\left(z^{(n)}\right)$. Therefore, the log complete likelihood is given by

$$L = \sum_{n=1}^{N} \{\ln \mathrm{Normal}\big(x^{(n)} \mid \mathrm{W} z^{(n)} + \mu, \epsilon \mathrm{I}_M \big) + \ln \mathrm{Normal}\big(z^{(n)} \mid 0, \mathrm{I}_d \big) \}$$

$$+ \sum_{l=1}^{d} \ln \mathrm{Normal}(\mathrm{w}_l \mid 0, \alpha_l \mathrm{I}_M),$$

$$= -\frac{1}{2} \sum_{n=1}^{N} \left\{ \frac{1}{\epsilon} \left\| x^{(n)} - \mathrm{W} z^{(n)} - \mu \right\|^2 + \left\| z^{(n)} \right\|^2 \right\} - \frac{1}{2} \sum_{l=1}^{d} \left\{ \frac{1}{\alpha_l} \|w_l\|^2 + M \ \ln \alpha_l \right\} + \text{constant}.$$

An exact Bayesian inference is intractable unless we resort to Monte Carlo methods, which is not a viable solution in our application scenario due to the interdependency between W and z. For a tractable and numerically stable solution, we apply the variational Bayes approximation[17] assuming $\epsilon \ll 1$, in which the posterior for W and z converges to the Dirac delta function. The entire training procedure is similar to that of the standard probabilistic principal component analysis[17]:

- Initialize: $\mathrm{A} = \mathrm{diag}(\alpha_1, \dots, \alpha_d), \mathrm{W}$. For all $(n, i) \in \mathcal{S}$, $x_i^{(\mathrm{n})} = 0$.
- Repeat until convergence:
  - $\bar{\mathrm{Z}} \leftarrow \left[\epsilon \mathrm{I}_d + \overline{\mathrm{W}}^\top \overline{\mathrm{W}} \right]^{-1} \overline{\mathrm{W}}^\top \Phi$
  - $\overline{\mathrm{W}} \leftarrow \Phi \bar{\mathrm{Z}}^\top \mathrm{A} [\bar{\mathrm{Z}} \bar{\mathrm{Z}}^\top \mathrm{A} + \epsilon \mathrm{I}_d]^{-1}$
  - $\mu \leftarrow \frac{1}{N} (\mathrm{X} - \overline{\mathrm{W}} \bar{\mathrm{Z}}) 1_N$
  - $\alpha_i = \frac{1}{M} \|\overline{w}_i\|^2$ for $i = 1, \dots, d$
  - $x_i^{(n)} \leftarrow \left[\mathrm{W} z^{(n)} + \mu \right]_i$ for all $(n, i) \in \mathcal{S}$.
- Output: $\overline{\mathrm{W}}, \mu$

Here, $\mathcal{S}$ is the set of missing entries in the training data set, $\bar{Z}$ and $\overline{\mathrm{W}}$ are the posterior means of Z and W, respectively, $\overline{w}_i$ is the i-th column vector of $\overline{\mathrm{W}}$, $\bar{z}^{(n)}$ is the n-th column vector of $\bar{\mathrm{Z}}$,

1_N denotes N-dimensional column vector of ones, and $\Phi = \mathrm{X} - \mu 1_N^\top$ with $\mathrm{X} = \left[x^{(1)}, \dots, x^{(N)}\right]$. Also, $[\cdot]_i$ denotes the i-th element of the vector in the parenthesis. In our implementation, convergence is declared if the relative error from the previous iteration round becomes less than 10^{-5}.

The proposed model was designed to enable automated selection of the latent dimension, d. With a sufficiently large d (we generally recommend setting $d = \min\{M, N\text{-}1\}\text{-}1$ for $M > 2$), it is often observed that many α_is end up being numerically zero upon convergence. Moreover, $\alpha_i = 0$ implies $w_i = 0$ since the prior distribution has a zero mean. In such a situation, the i-th latent dimension does not actively contribute to the model. In this manner, the model automatically chooses a sufficient number of latent dimensions. This is an example of the ARD mechanism of Bayesian learning.

Now let us consider how to perform data imputation for a test data set. Suppose that we are given a test data set $\{x^{[1]}, \dots, x^{[N']}\}$, where N' is the number of test samples. We used the square bracket to denote the sample index not to be confused with the training samples. In addition, let $\mathcal{S}'$ be the set of missing entries of the test data set. For imputation, we use $\overline{\mathrm{W}}, \mu$ learned from the training data to perform the following iterative procedure:

- Initialize: For all $(n, i) \in \mathcal{S}'$, $x_i^{[n]} = \mu_i$.
- Repeat until convergence:
  - Find $z^{[n]}$ that satisfy $[\epsilon \mathrm{I}_d + \overline{\mathrm{W}}^\top \overline{\mathrm{W}}] z^{[n]} = \overline{\mathrm{W}}^\top \left(x^{[n]} - \mu\right)$ for $n = 1, \dots, N'$.
  - Update ${x_j}^{[n]} \leftarrow \left[\overline{\mathrm{W}} z^{[n]} + \mu\right]_j$ for all $(n, j) \in \mathcal{S}'$.

The first line of the iterative part requires solving a d-dimensional simultaneous linear equation. It should be noted that $\overline{\mathrm{W}}$ maps a test sample onto the latent subspace learned from the training data. This can be interpreted as *domain adaptation* from the source domain represented by the training data to the target domain represented by the test data.

Code availability

For the classification module, we used Python's standard open-source library: LR and RF are based on the corresponding function in scikit-learn 1.0. DL is based on Keras 2.4.0.

For the adaptation module, we developed our own implementation in Python using NumPy 1.19.5. The code has been available on GitHub (https://github.com/Idesan/bpca).

References

1. Shimamoto K, Ando K, Fujita T, et al. The Japanese Society of Hypertension Guidelines for the Management of Hypertension (JSH 2014). *Hypertens Res*. Apr 2014;37(4):253-390. doi:10.1038/hr.2014.20
2. Satoh F, Morimoto R, Seiji K, et al. Is there a role for segmental adrenal venous sampling and adrenal sparing surgery in patients with primary aldosteronism? *Eur J Endocrinol*. Oct 2015;173(4):465-77. doi:10.1530/EJE-14-1161
3. Kitamoto T, Kitamoto KK, Omura M, et al. Precise Mapping of Intra-Adrenal Aldosterone Activities Provides a Novel Surgical Strategy for Primary Aldosteronism. *Hypertension*. Sep 2020;76(3):976-984. doi:10.1161/HYPERTENSIONAHA.119.14341
4. Wada N, Miyoshi A, Usubuchi H, et al. Prediction of unilateral hyperaldosteronism on adrenal vein sampling using captopril challenge test in patients with primary aldosteronism. *Endocrine Journal*. 2021;68(1):45-51. doi:10.1507/endocrj.ej20-0329
5. Nishikawa T, Omura M, Satoh F, et al. Guidelines for the diagnosis and treatment of primary aldosteronism--the Japan Endocrine Society 2009. *Endocr J*. 2011;58(9):711-21.
6. Funder JW, Carey RM, Mantero F, et al. The Management of Primary Aldosteronism: Case Detection, Diagnosis, and Treatment: An Endocrine Society Clinical Practice Guideline. *J Clin Endocrinol Metab*. May 2016;101(5):1889-916. doi:10.1210/jc.2015-4061
7. Rossi GP, Auchus RJ, Brown M, et al. An Expert Consensus Statement on Use of Adrenal Vein Sampling for the Subtyping of Primary AldosteronismNovelty and Significance. *Hypertension*. 2014;63(1):151-160.
8. Kitamoto T, Omura M, Suematsu S, Saito J, Nishikawa T. KCNJ5 mutation as a predictor for resolution of hypertension after surgical treatment of aldosterone-producing adenoma. *J Hypertens*. Mar 2018;36(3):619-627. doi:10.1097/HJH.0000000000001578
9. Nanba K, Yamazaki Y, Bick N, et al. Prevalence of Somatic Mutations in Aldosterone-Producing Adenomas in Japanese Patients. *J Clin Endocrinol Metab*. Nov 1 2020;105(11):e4066-e4073. doi:10.1210/clinem/dgaa595
10. Williams TA, Lenders JWM, Mulatero P, et al. Outcomes after adrenalectomy for unilateral primary aldosteronism: an international consensus on outcome measures and analysis of remission rates in an international cohort. *Lancet Diabetes Endocrinol*. Sep 2017;5(9):689-699. doi:10.1016/S2213-8587(17)30135-3
11. James PA, Oparil S, Carter BL, et al. 2014 evidence-based guideline for the management of high blood pressure in adults: report from the panel members appointed to the Eighth Joint National Committee (JNC 8). *JAMA*. Feb 5 2014;311(5):507-20. doi:10.1001/jama.2013.284427
12. Shmueli G. To Explain or to Predict? *Statistical Science*. 2010;25(3):289-310, 22.
13. Ilicic T, Kim JK, Kolodziejczyk AA, et al. Classification of low quality cells from single-cell RNA-seq data. *Genome Biology*. 2016/02/17 2016;17(1):29. doi:10.1186/s13059-016-0888-1
14. Hastie T, Tibshirani R, Friedman J. The Elements of Statistical Learning, 2nd ed. 2009, Springer. *New York*.1-282.
15. Oba S, Sato MA, Takemasa I, Monden M, Matsubara K, Ishii S. A Bayesian missing value estimation method for gene expression profile data. *Bioinformatics*. Nov 1 2003;19(16):2088-96. doi:10.1093/bioinformatics/btg287
16. Brock GN, Shaffer JR, Blakesley RE, Lotz MJ, Tseng GC. Which missing value imputation method to use in expression profiles: a comparative study and two selection schemes. *BMC Bioinformatics*. Jan 10 2008;9(1):12. doi:10.1186/1471-2105-9-12
17. Bishop CM. Pattern recognition and machine learning. *Springer*. 2006;1:738.

Table S1. Comparison of clinical characteristics in Sapporo related to Table 1.

	Unilateral PA	Bilateral PA		
	**Surgery**	**Surgery**	**Medication**	
Variables	(n = 15)	(n = 1)	(n = 41)	P value
Age (yr)	49.3 ± 12.9	47	49.3 ± 11.3	0.9814
Sex (male/female)	11 / 4	Female	13 / 28	0.0139
BMI	25.9 ± 3.7	23.7	25.5 ± 4.4	0.8567
SBP (mmHg)	144 ± 14.3	126	139.6 ± 17.1	0.4571
DBP (mmHg)	85.7 ± 12.5	82	85.4 ± 9.3	0.9413
Known duration of hypertension	6 (3 - 10)	2	2.5 (0.5 - 4)	0.0199
Antihypertension medication (defined daily dose)	1.9 (1 - 3.4)	2	1 (0.3 - 1.3)	0.0079
Plasma aldosterone (ng/dl)	37.3 (22.3 - 65.4)	41.1	16.2 (11.4 - 19.5)	<.0001
Plasma renin activity (ng/ml/hr)	0.2 (0.1 - 0.3)	0.3	0.4 (0.2 - 0.5)	0.0478
Lowest serum potassium ion concentration (mmol/L)	3.1 ± 0.7	3	3.7 ± 0.4	0.0073
Creatinine (mmol/L)	0.8 ± 0.2	0.68	0.7 ± 0.2	0.2652
eGFR (mL/min per 1.73m2)	83.5 ± 24.3	72.4	82.5 ± 15.6	0.8386
Adrenal nodule (mm)*	11.5 ± 6.9	8	0 ± 0	N.A.
Laterality of surgical side (right/left)	7 / 8	1 / 0	N.A.	N.A.
Laterality of Image positive side (right/left/bilateral/undetectable)	6 / 7 / 1 / 1	1 / 0 / 0 / 0	2 / 6 / 2 / 31	N.A.

Diagnostic outcome of cAVS (right/left/bilateral/failed)	7 / 8 / 0 / 0	0 / 0 / 0 / 1	18 / 5 / 18 / 0	< 0.0001
L.I. in cAVS	22.3 (6.8 - 44.4)	N.A.	2.4 (1.5 - 7.2)	< 0.0001
Diagnostic outcome of ACTH-cAVS (right/left/bilateral/failed)	7 / 8 / 0 / 0	Bilateral	0 / 0 / 41 / 0	N.A.
L.I. in ACTH-cAVS	22.6 (6 - 37)	2.2	1.3 (1.1 - 1.6)	< 0.0001
SI_RAV	5 (2.7 - 19.6)	N.A.	7.3 (2.6 - 22.8)	0.0619
SI_LAV	21.9 (8.6 - 50.9)	N.A.	17.7 (7.5 - 56.4)	0.8255
Biochemical outcome (Complete/Partial/Absent) [Complete (%)]	12 / 1 / 2 [80.0%]	Partial	N.A.	N.A.
Clinical outcome (Complete/Partial/Absent) [Complete (%)]	5 / 6 / 4 [33.3%]	Complete	N.A.	N.A.

The clinical characteristics, diagnostic outcome, and post-surgical outcome of Sapporo cohort. The data presentation and abbreviations are show in the same way as Table 1.

Table S2. Comparison of clinical characteristics in Sendai related to Table 1.

	Unilateral PA	Bilateral PA		
	**Surgery**	**Surgery**	**Medication**	
Variables	(*n* = 33)	(*n* = 1)	(*n* = 39)	*P* value
Age (yr)	56 ± 11.6	65	51.2 ± 11.7	0.1437
Sex (male/female)	20 / 13	Female	17 / 22	0.2109
BMI	23.3 ± 3.5	19.6	24.9 ± 3.7	0.0804
SBP (mmHg)	150.2 ± 17.8	143	148.7 ± 20.1	0.8925
DBP (mmHg)	95.6 ± 12.2	87	96.8 ± 13.5	0.7177
Known duration of hypertension	16 (6 - 19)	27	5 (1 - 11)	0.0023*
Antihypertension medication (defined daily dose)	2.7 (1.2 - 3.7)	1.83	1.3 (0.5 - 2)	0.0111*
Plasma aldosterone (ng/dl)	42.8 (27 - 62.1)	36.1	24 (17.7 - 30.4)	<.0001*
Plasma renin activity (ng/ml/hr)	0.2 (0.2 - 0.4)	0.2	0.3 (0.2 - 0.4)	0.2671
Lowest serum potassium ion concentration (mmol/L)	3.1 ± 0.5	3	3.6 ± 0.4	<.0001*
Creatinine (mmol/L)	0.9 ± 0.3	0.87	0.8 ± 0.4	0.2537
eGFR (mL/min per 1.73m2)	67.3 ± 22.2	50.6	79.2 ± 20.2	0.0376*
Adrenal nodule (mm)*	15.2 ± 8	10	4.3 ± 7.8	<.0001*
Laterality of surgical side (right/left)	16 / 17	1 / 0	N.A.	N.A.
Laterality of Image positive side (right/left/bilateral/undetectable)	12 / 15 / 3 / 3	1 / 0 / 0 / 0	5 / 4 / 1 / 29	N.A.

Diagnostic outcome of cAVS (right/left/bilateral/failed)	14 / 14 / 3 / 2	1 / 0 / 0 / 0	16 / 2 / 18 / 3	0.0016
L.I. in cAVS	17.9 (5.4 - 37)	4.5	2.3 (1.3 - 4.6)	< 0.0001
Diagnostic outcome of ACTH-cAVS (right/left/bilateral/failed)	12 / 13 / 7 / 1	1 / 0 / 0 / 0	0 / 0 / 39 / 0	N.A.
L.I. in ACTH-cAVS	10.5 (6.4 - 21.9)	6.0	1.6 (1.1 - 2.1)	< 0.0001
SI_RAV	3.7 (1.8 - 5.9)	4.7	3.4 (2 - 5.2)	0.837
SI_LAV	2.8 (2.1 - 4.5)	2.5	2.9 (2.4 - 5.5)	0.7022
Biochemical outcome (Complete/Partial/Absent) [Complete (%)]	33 / 0 [100.0%]	Partial	N.A.	N.A.
Clinical outcome (Complete/Partial/Absent) [Complete (%)]	10 / 21 / 2 [30.3%]	Partial	N.A.	N.A.

The clinical characteristics, diagnostic outcome, and post-surgical outcome of Sendai cohort. The data presentation and abbreviations are show in the same way as Table 1.

Table S3. Comparison of clinical characteristics in Yokohama related to Table 1.

	Unilateral PA	Bilateral PA		
	**Surgery**	**Surgery**	**Medication**	
Variables	(n = 41)	(n = 11)	(n = 28)	P value
Age (yr)	49.5 ± 9.3	50.5 ± 12.2	49 ± 10.5	0.9183
Sex (male/female)	20 / 21	6 / 5	10 / 18	0.4455
BMI	24.2 ± 3.5	25 ± 3.7	24.7 ± 3.7	0.7604
SBP (mmHg)	138.8 ± 14.8	142.2 ± 19.6	136.9 ± 20.1	0.6975
DBP (mmHg)	86.1 ± 13.1	83.2 ± 7.9	85.6 ± 10.3	0.7594
Known duration of hypertension	8 (3 - 15)†	8 (5 - 12)‡	2.5 (1.3 - 5)†‡	0.0015
Antihypertension medication (defined daily dose)	2 (1 - 2.1)†	1.4 (1 - 2.7)	1 (0.3 - 1.5)†	0.0071
Plasma aldosterone (ng/dl)	30.5 (18.4 - 50.7)†	43.5 (21.8 - 51)‡	15.6 (12.8 - 19.2)†‡	0.0001
Plasma renin activity (ng/ml/hr)	0.2 (0.1 - 0.3)	0.5 (0.2 - 0.6)	0.3 (0.1 - 0.6)	0.1039
Lowest serum potassium ion concentration (mmol/L)	3.3 ± 0.5†	3.3 ± 0.4‡	3.8 ± 0.2†‡	0.0003
Creatinine (mmol/L)	0.7 ± 0.2	0.7 ± 0.2	0.7 ± 0.1	0.8608
eGFR (mL/min per 1.73m2)	85.4 ± 20.9	84.1 ± 19.6	82.3 ± 16.8	0.8148
Adrenal nodule (mm)*	15 ± 7.0†	14.2 ± 7.4‡	4.9 ± 6.6†‡	< 0.0001
Laterality of surgical side (right/left)	21 / 20	8 / 3	N.A.	N.A.
Laterality of Image positive side (right/left/bilateral/undetectable)	12 / 17 / 10 / 2	3 / 2 / 5 / 1	0 / 6 / 6 / 16	< 0.0001

Diagnostic outcome of cAVS (right/left/bilateral/failed)	17 / 17 / 6 / 1	7 / 3 / 1 / 0	7 / 6 / 15 / 0	0.0091
L.I. in cAVS	16.4 (2.6 - 32.3)†	13.7 (2.3 - 18)‡	1.9 (1.3 - 2.6)†‡	< 0.0001
Diagnostic outcome of ACTH-cAVS (right/left/bilateral/failed)	15 / 14 / 11 / 1	3 / 3 / 5 / 0	0 / 0 / 28 / 0	< 0.0001
L.I. in ACTH-cAVS	8.8 (3.4 - 23.6)†	4.2 (1.5 - 8.0)‡	1.4 (1.2 - 1.7)†‡	< 0.0001
SI_RAV	10.5 (4.1 - 20.4)	15.5 (2.0 - 31.6)	12.6 (3.3 - 20.5)	0.9157
SI_LAV	20.8 (9.5 - 39.4)	18.8 (8.1 - 31.9)	30.4 (13.8 - 49.8)	0.1358
Biochemical outcome (Complete/Partial/Absent) [Complete (%)]	37 / 4 / 0 [90.2%]	7 / 4 / 0 [63.6%]	N.A.	N.A.
Clinical outcome (Complete/Partial/Absent) [Complete (%)]	15 / 26 / 0 [36.6%]	1 / 10 / 0 [9.1%]	N.A.	N.A.

The clinical characteristics, diagnostic outcome, and post-surgical outcome of Yokohama cohort. The data presentation and abbreviations are show in the same way as Table 1.

Table S4. The concordance of diagnostic outcomes among sAVS, cAVS before and after ACTH stimulation, and CT imaging.

		CT imaging	cAVS (LI > 2.0)	ACTH-cAVS (LI > 4.0)
sAVS	Right	0.66	0.93	0.81
	Left	0.82	0.84	0.78
	Bilateral	0.75	0.44	0.94
	Number of consistent diagnosis with sAVS [n (%)]	157 (74.8%)	128 (63.1%)	183 (88.0%)
	Total number of cases	210	203	208

The concordance proportion was calculated by counting the same diagnostic outcome in each patient in each pair. sAVS diagnosed right or left PA in 44 and 45 cases, and bilateral in 121 cases. The consistent locational diagnosis were seen in 38, 38, and 52 cases in right, left, or bilateral by CT imaging, in 29, 37, and 14 cases by cAVS, and in 34, 35, and 114 cases by ACTH-cAVS. cAVS: conventional adrenal venous sampling, ACTH: adrenocorticotropic hormone, sAVS: segmental selective AVS, LI: lateralization index

Table S5. Post-operative clinical parameters shown in Table 2.

		Surgery-track	AVS recommend	
Post-operative data	*N*	**84**	**18**	*P* value
Follow up term (month)	102	11 (11 - 12)	12 (9.8 - 13)	0.2049
Antihypertension medication (defined daily dose)	102	0.5 (0 - 2)	1 (0 - 2)	0.5796
SBP (mmHg)	100	124.9 ± 12.7	129.4 ± 11.6	0.1815
DBP (mmHg)	100	81.8 ± 10.7	84.2 ± 8.7	0.3831
Plasma aldosterone (ng/dl)	102	8.7 (6.1 - 11.9)	10.1 (8.5 - 12.7)	0.1806
Plasma renin activity (ng/ml/hr)	102	1.1 (0.5 - 2.2)	1.1 (0.8 - 2.1)	0.5474
Serum cortisol (µg/dl)	81	8.6 (6.2 - 11.2)	7.2 (5.6 - 12.7)	0.852
Serum potassium (mEq/L)	102	4.3 ± 0.4	4.5 ± 0.5	***0.0208***
Serum creatinine (mg/dl)	102	1 ± 0.4	1 ± 0.3	0.5225
eGFR (mL/min/1.73m^2)	102	65.5 ± 21.4	61.1 ± 16.5	0.4219
Biological outcome (Complete/Partial/Absent) [Complete (%)]	102	72 / 11 / 1 [85.7%]	17 / 0 / 1 [94.4%]	0.0645
Clinical outcome (Complete/Partial/Absent) [Complete (%)]	102	29 / 52 / 3 [34.5%]	3 / 12 / 3 [16.7%]	0.0528

The post-surgical clinical characteristics and outcome in sAVS bypass and recommend group. The data presentation and abbreviations are show in the same way as Table 2.

Table S6. Primer pairs used for mutation detection by Sanger sequencing

Gene	Exon		Primers	Amplicon (bp)	RefSeq
KCNJ5	2	Forward	ACCTGGACCATGTTGGCGACC	286	NM_000890.3
	2	Reverse	TCCCGCATGGAGATGACTGCG		
	2	Forward	CGACCAAGAGTGGATTCCTT	234	NM_000890.5
	2	Reverse	AGGGTCTCCGCTCTCTTCTT		
ATP1A1	3	Forward	GCCGGGGATTAACATCTGCT	399	NM_000701.7
	6	Reverse	CTTCCACCAGATCCCCAACC		
ATP1A1	7	Forward	AATTGTGTTGAAGGCACCGC	362	NM_000701.7
	9	Reverse	CCCAAGGTCTCCACAGCTTC		
ATP2B3	5	Forward	TAAGAAAGGCAAGCAGCAGGA	410	NM_021949.3
	8	Reverse	AGATGGTGACAGCAAGAGGC		
CACNA1D	8	Forward	TGCGATAGGATGGGAATGGC	217	NM_000720.3
	9	Reverse	GTCCTCAGCTTGGGTGATCC		
CACNA1D	8	Forward	GGATTTGAATTGCCCTGGGTG	204	NM_001128840.2
	9	Reverse	AGCTTGGGTGATCCAATCCA		
CACNA1D	15	Forward	CCAGATCCTGACAGGCGAAG	312	NM_000720.3
	19	Reverse	ACCTTGTTGTCACTGTTGGC		
CTNNB1	1	Forward	GACGGAGGAAGGTCTGAGGA	530	NM_001904.3

4	Reverse	TTCAGCCAAACGCTGGACAT

Table S7. Summary of the classifiers trained.

	Objective	*Algorithm*	*Hyperparameter*	*Range*
LR	Log likelihood	IRLS	C (for either ‘l1’ or ‘l2)	$0.001 \leq C \leq 6$
RF	Gini index	Axis-wise partition	nTrees	$50 \leq \text{nTrees} \leq 2000$
DL	Binary cross entropy	Stochastic gradient	Nh0, Nh1, batch	$6 \leq Nh0, Nh1 \leq 16,\ 4 \leq \text{batch} \leq\ 16$

The Algorithm is to optimize the objective function on the training dataset, given a set of hyperparameters. The hyperparameters are optimized against the f-score evaluated on the validation dataset.

Table S8. Clinical characteristic of the cases mis-classified into Surgery-track

Project Case ID	**Case#10**	**Case#45**	**Case#115**	**Case#157**
Institution	Yokohama	Yokohama	Tohoku	Sapporo
Age (yr)	50	51	53	52
Sex (male/female)	Male	Male	Female	Female
Antihypertension medication (defined daily dose)	1.5	1.3	4.5	1.3
SBP (mmHg)	150	165	180	152
DBP (mmHg)	92	98	97	90
Lowest serum potassium ion concentration (mmol/L)	2.3	3	1.9	3.3
Ptassium replacement therapy	(+)	(+)	(+)	(+)
Serum creatinine (mg/dl)	0.82	0.92	2.56	0.54
eGFR (mL/min/1.73m^2)	78.43	68.76	16.4045557	90.5
Plasma aldosterone (ng/dl)	80.9	81.2	150.2	23
Plasma renin activity (ng/ml/hr)	0.1	0.4	0.2	0.1
Plasma cortisol (μg/dl)	7.1	7.8	13.7	8.2
Plasma cortisol after DST (μg/dl)	0.5	0.8	4.8	1.2
ARR after CCT	461	246	497	235
Plasma aldosterone after AST (ng/dl)	127	108	235.7	68.2
Plasma cortisol after AST (μg/dl)	24.5	21.5	25.3	30.8
Laterality of Image positive side (right/left/bilateral/undetectable)	Bilateral	Bilateral	Bilateral	Left
Adrenal nodule (Right/Left) (mm)	(16, 5)	(18, 9)	(23, 25)	(0, 14)
L.I. in cAVS	17.6	19.9	1.1	2.2
Diagnostic outcome of cAVS (right/left/bilateral/failed)	Left	Left	Bilateral	Left
L.I. in ACTH-cAVS	10.7	8.0	1.9	2.1
Diagnostic outcome of ACTH-cAVS (right/left/bilateral/failed)	Left	Left	Bilateral	Bilateral

Diagnostic outcome of ssAVS (right/left/bilateral/failed)	Left	Bilateral	Bilateral	Bilateral
Laterality of surgical side (right/left)	Left	Left	Medication	Medication
Biochemical outcome (Complete/Partial/Absent)	Complete	Complete	n.a.	n.a.
Clinical outcome (Complete/Partial/Absent)	Partial	Partial	n.a.	n.a.

The clinical characteristics of four cases that were misclassified to recommend surgery through the diagnostic flow chart shown in Figure 3. Case#10 had a smaller tumor responsible for primary aldosteronism, and resection of the tumor resulted in a complete biochemical cure. Case#45 was diagnosed as bilateral aldosterone-producing adenomas; however, the left side showed dominant aldosterone secretion. Case#115 showed bilateral tumors and was diagnosed as idiopathic hyperaldosteronism by sAVS. Case#157 showed a left-sided tumor but was diagnosed as bilateral aldosteronism by sAVS. Thus, Case#115 and Case#157 were medically treated.

Figure S1. Data imputation of single dataset and transfer learning between two datasets

A

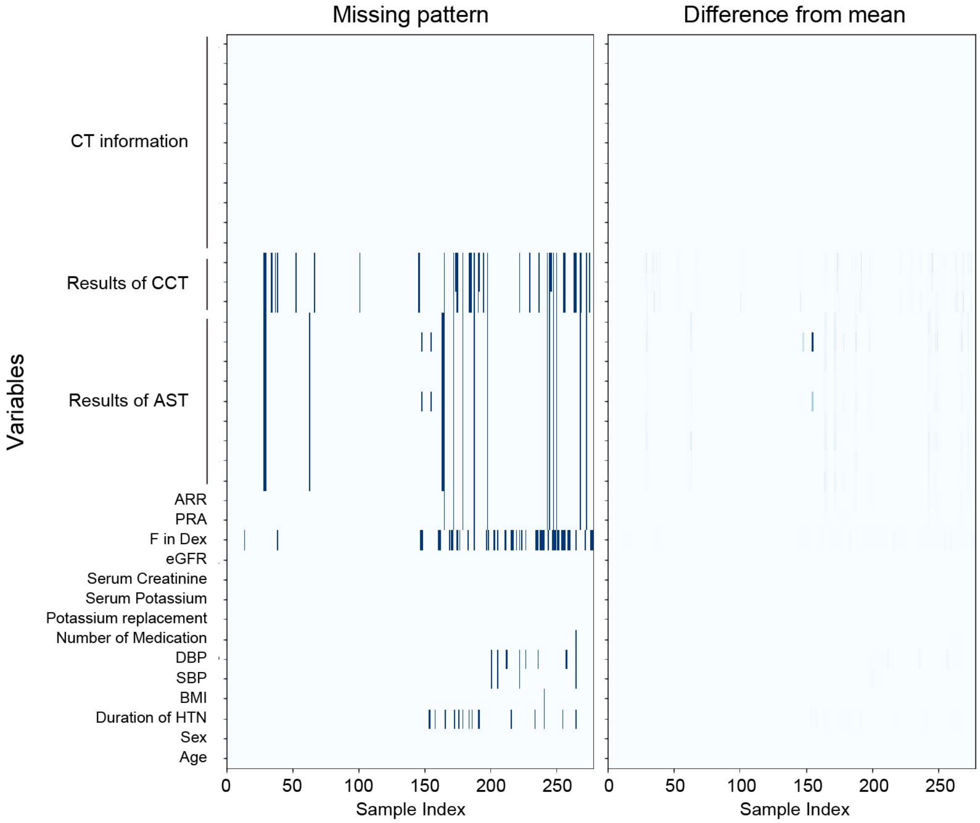

B

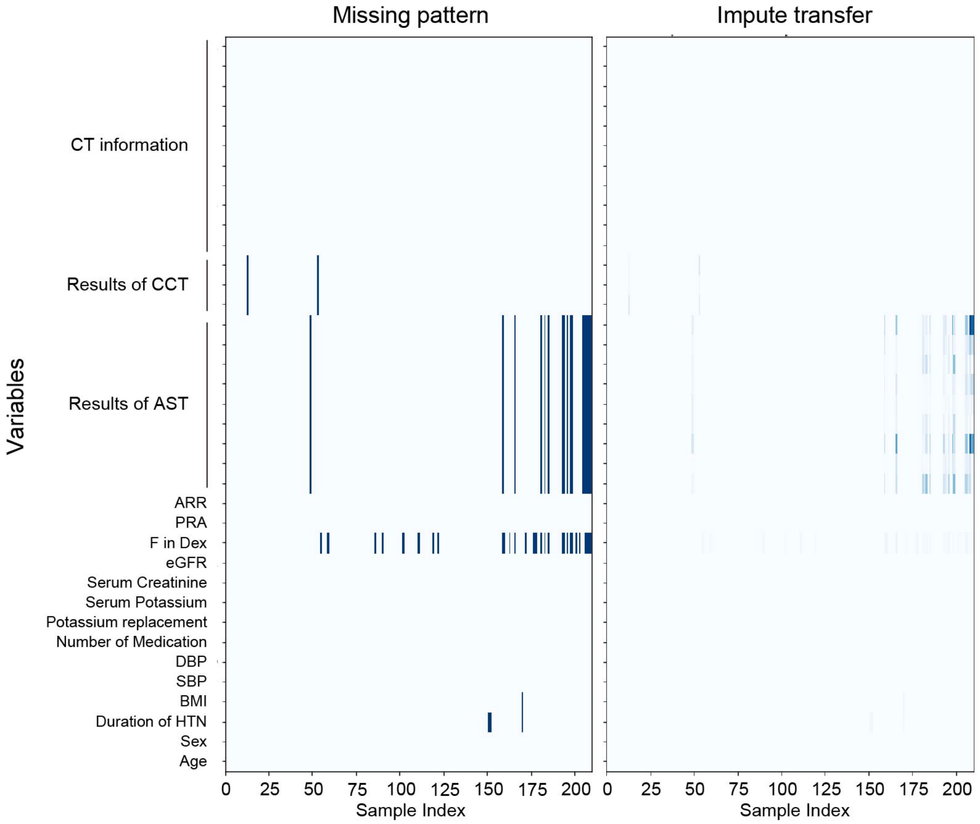

(A) The data imputation of dataset from single center cohort (*Hypertension 2020; 76(3): 976-84*) is shown. Variables are in vertical axis and sample index for each case is shown in horizontal axis. This heat map is demonstrating missing data (left panel) or difference from mean value in each variable after data imputation (right panel) in each case. shown as sample index. (B) The data imputation developed in (A) is applied for multi-center cohort (transfer learning). Data presentation is in the same way in (A). Right panel shows the data after data imputa transfer. Data units are used in the same way in Table 2. ARR, aldosterone-to-renin ratio; PAC, plasma aldosterone concentration; eGFR, estimated glomerular filtration rate; CCT, captopril challenge test; F, free plasma cortisol; PRA, plasma renin activity; AST, ACTH infusion test; DST, dexamethasone suppression test; SBP, systolic blood pressure; DBP, diastolic pressure; HTN, hypertension; BMI, body mass index.

Figure S2. Breakdown list of the coefficients from logistic regression model to detect the cases on the surgery-track

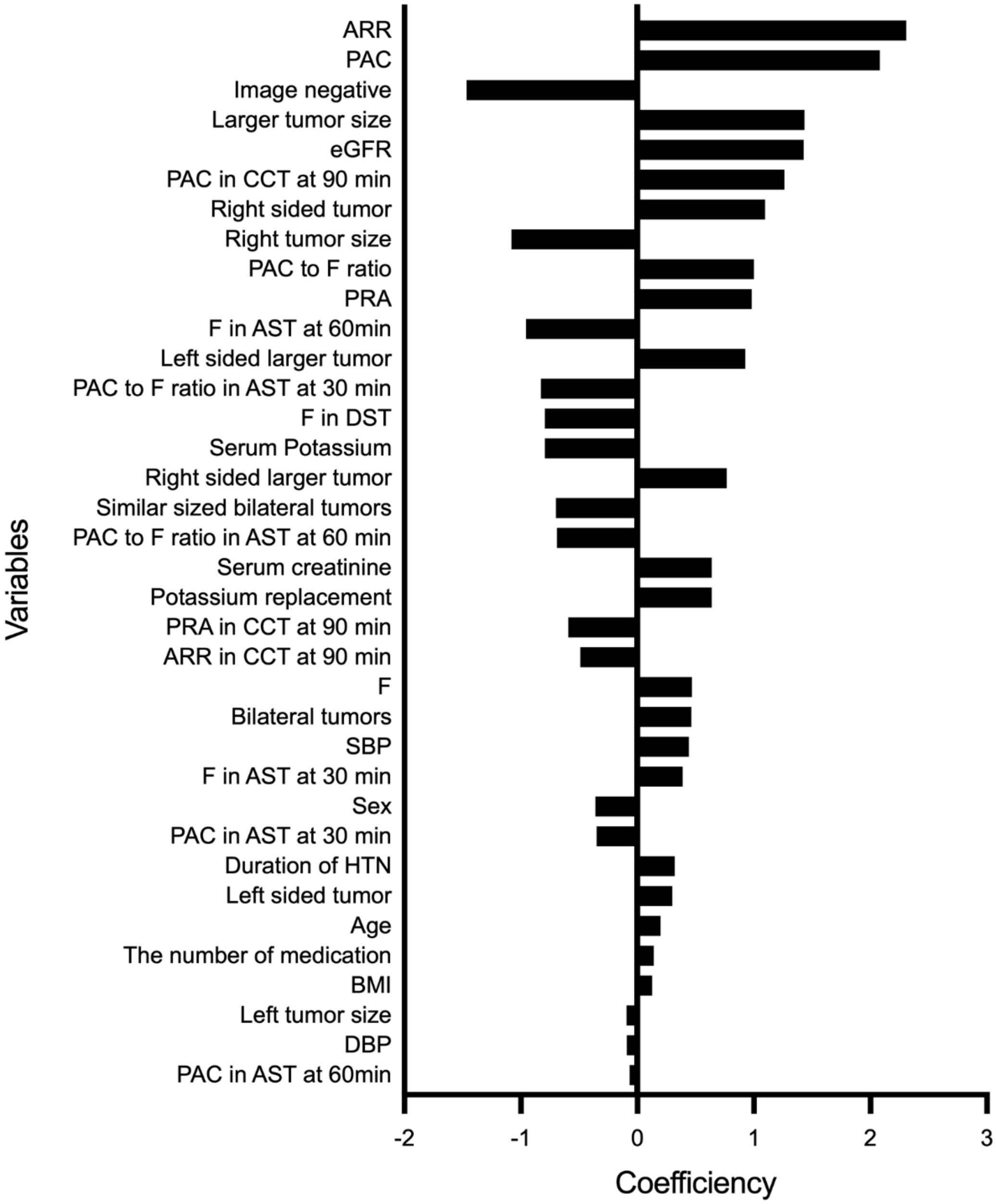

The coefficients from logistic regression model to detect the cases bypassing AVS is shown. Data units and abbreviations are expressed in the same manner in Figure S1.

Figure S3. Breakdown list of the coefficients from logistic regression model to detect the cases on the medication-track

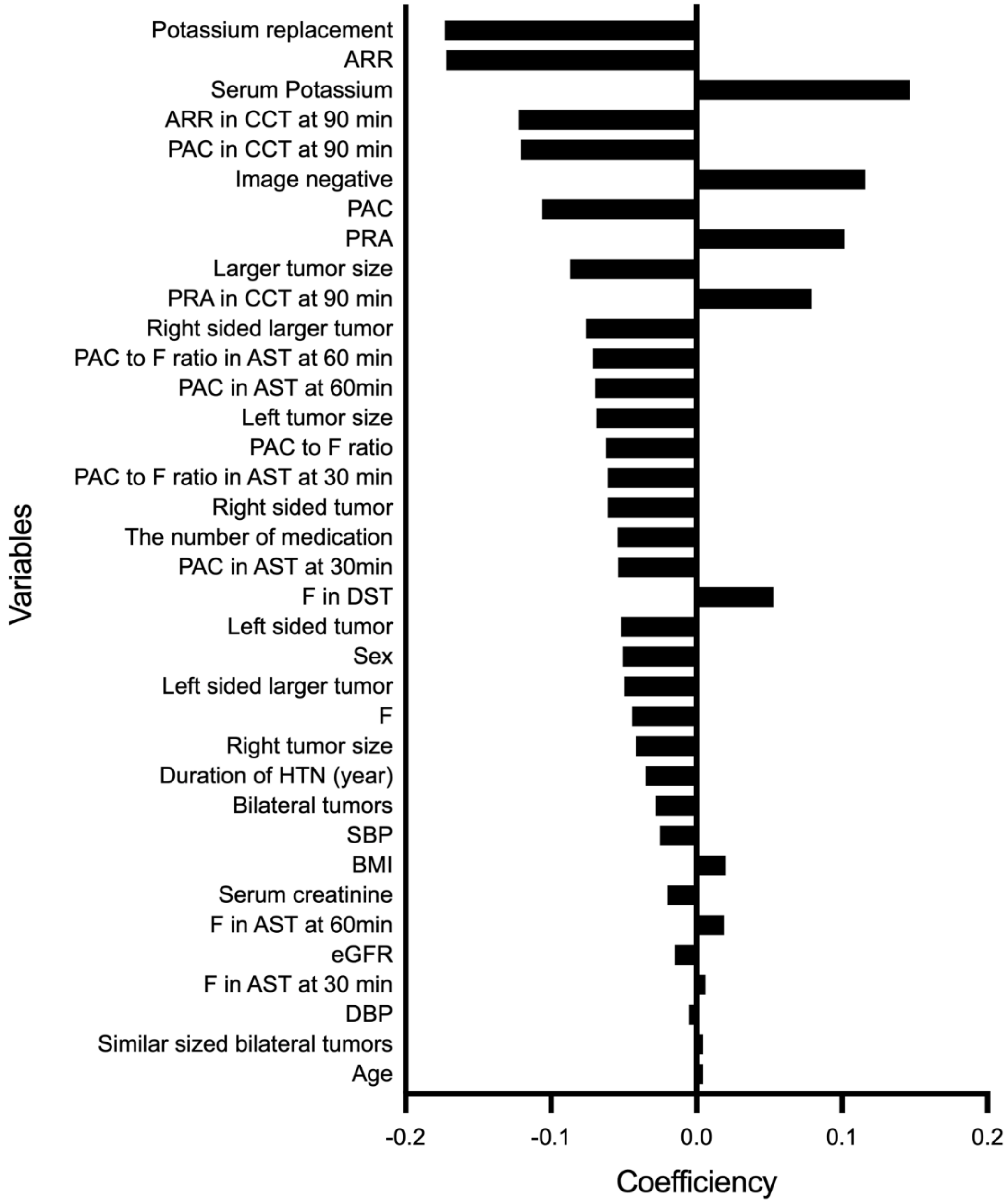

The coefficients from logistic regression model to detect the cases of IHA is shown. The data units and abbreviations are expressed in the same manner in Figure S1.
